# Pediatric Bilateral Lumbosacral Dislocation Without Fracture: An Exemplary Case and Literature Review

**DOI:** 10.7759/cureus.30417

**Published:** 2022-10-18

**Authors:** Gabrielle Santangelo, Sandra Catanzaro, Nicholas Contento, Redi Rahmani, Howard Silberstein

**Affiliations:** 1 Neurological Surgery, University of Rochester Medical Center, Rochester, USA; 2 Neurosurgery, University of Rochester, Rochester, USA; 3 Neurological Surgery, University of Rochester School of Medicine and Dentistry, Rochester, USA; 4 Neurosurgery, University of Rochester Medical Center, Rochester, USA

**Keywords:** pediatric spine, facet dislocation, lumbosacral, pediatric facet dislocation, lumbrosacral dislocation

## Abstract

Lumbosacral facet dislocations are rare lesions typically seen in high-energy trauma. This type of injury is a severe flexion-distraction injury and is extremely rare, with only six other documented reports. A recent case series proposed a classification for lumbosacral injuries which would classify the present case as a 1C, meaning a bilateral dislocation with anterior slippage of the L5 vertebra without fractures of the articulating processes (AP), pars interarticularis (PI), or vertebral bodies (VB). In this case report, we discuss the third case of a class 1C injury in a pediatric patient, review the associated literature and discuss the presentation, diagnosis, management, and prognosis of these rare dislocations.

## Introduction

Lumbosacral facet dislocations are rare injuries typically associated with high-energy trauma [1-4]. This type of injury is a severe flexion-distraction injury between vertebrae that occurs when the inferior articulating process of the superior vertebra is displaced anteriorly from the superior articular process of the inferior vertebra. Even rarer is a lumbosacral dislocation injury without an accompanying fracture of the pars interarticularis, articulating processes, or vertebral bodies-classified as a 1C injury [5]. A literature review revealed six previously reported cases of class 1C lumbosacral injuries [1-4,6,7]. All six cases reported abnormal neurologic examination findings as a part of the patient presentation and only two documented cases involved the pediatric population. This case report will present the seventh overall, third pediatric, and first case without neurologic deficits in the literature. It will include a detailed report of a 16-year-old female who suffered from bilateral anterior dislocation of L5 on S1 after a motor vehicle accident and will compare to the previous reports of class 1C lumbosacral dislocations in regard to patient presentation, imaging findings, and surgical management.

## Case presentation

Patient presentation

A 16-year-old girl with a past medical history notable for pyelonephritis presented as a restrained driver in a motor vehicle accident associated with deployment of airbags. She was able to self-extricate from the vehicle but had significant low back pain. She was transported by emergency medical services to the emergency department for trauma evaluation and remained hemodynamically stable. During her trauma evaluation, she had no weakness, paresthesias, bowel or bladder incontinence or saddle anesthesia. Neurosurgical consultation was requested and she had no motor or sensory deficits and had normal reflexes on neurologic examination. 

**Figure 1:**
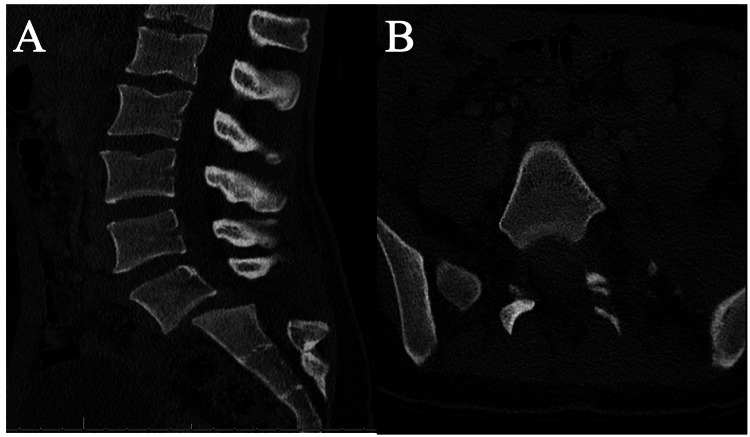
Pre-operative CT of the lumbar spine (A) Sagittal view of lumbar CT scan revealing grade 2 L5 on S1 traumatic anterior spondylolisthesis and (B) axial view of lumbar CT scan showing the S1 vertebral body and the bilateral L5 inferior articular processes in an abnormal ventral position compared to the S1 superior articular processes due to traumatic dislocation.

**Figure 2:**
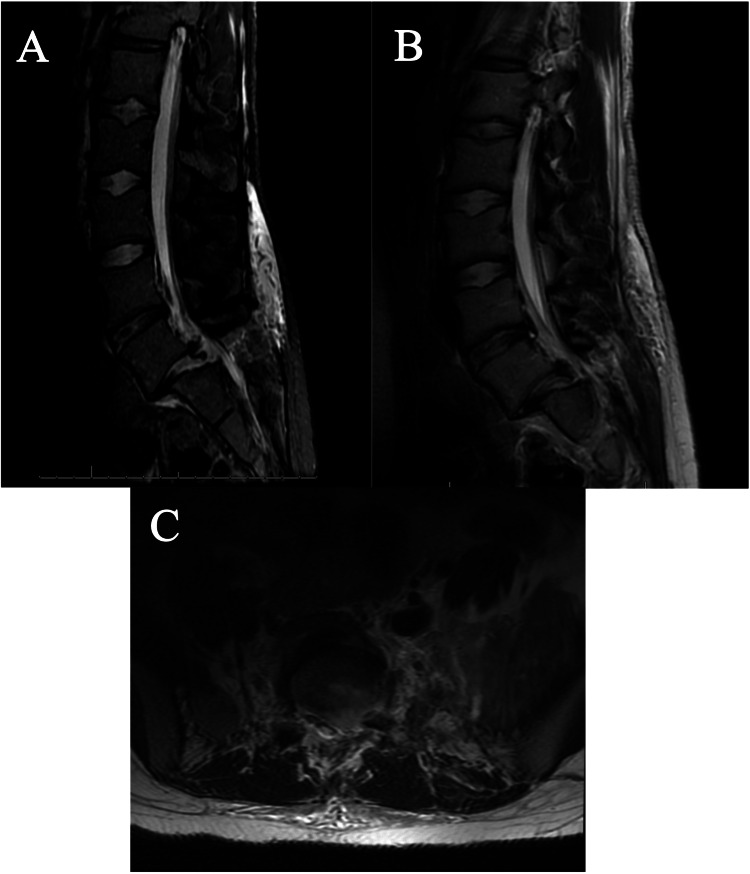
Pre-operative MRI of the lumbar spine (A) Sagittal view of lumbar MRI STIR sequence revealing L5 on S1 traumatic anterior spondylolisthesis with disruption of the PLL and extensive edema within the posterior ligamentous complex with associated widening of the L5/S1 interspinous space representing ligamentous disruption, (B) sagittal view of lumbar MRI T2 sequence again revealing L5 on S1 traumatic anterior spondylolisthesis with disruption of the PLL and posterior ligamentous complex in additional L5/S1 spinal canal stenosis, and (C) axial view of lumbar MRI T2 sequence showing the vertebral body and superior articular processes of S1 without the L5 inferior articular processes due to traumatic dislocation causing severe spinal stenosis.

Computed tomography imaging demonstrated left L5-S1 jumped facets and right-sided L5-S1 perched facets with grade 2 spondylolisthesis, measuring 1.5 cm (Figures 1A, 1B). There was an associated L5 transverse process fracture but no pars interarticularis, vertebral body, or articulating process fractures. Magnetic resonance imaging recapitulated these findings with evidence of severe canal stenosis at the L5-S1 level and posterior longitudinal ligament and interspinous ligament disruption (Figures 2A-2C).

Operative procedure and intra-operative findings

**Figure 3:**
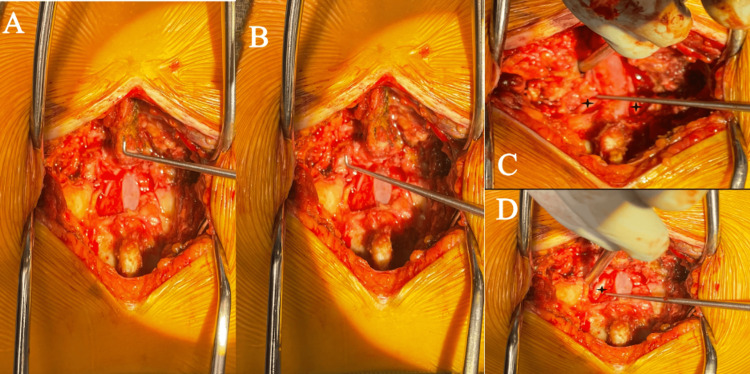
Intra-operative view of the lumbrosacral region (A) Laminectomy performed with L4 SP shown for orientation; (B) instrument pointing to the left L4/5 facet joint overlying the S1 facet; (C) nerve hook pulling thecal sac aside to show large, herniated disk with bilateral S1 nerve roots identified; and (D) star depicting the freed S1 nerve root.

She was taken to the operating room for L5-S1 laminectomy and instrumented fusion 12 hours after the presentation. A Wilson frame was used. There was no evidence of traumatic cerebrospinal fluid leak. A wide laminectomy and facetectomy were performed at the L5 and S1 levels to achieve reduction of the jumped facets (Figures 3A-3D). There was a large, herniated disk at the aforementioned levels with significant associated impingement of the thecal sac which was removed. 6.5 x 45mm screws were placed bilaterally at the L5 pedicles and 6.5 x 35 mm screws were placed bilaterally at the S1 level. An interbody device was not used as the authors felt that she had adequate anterior column support given her age and the robust nature of the remaining disk. To achieve posterolateral fusion along the facets, an autograft was placed on the decorticated bone surfaces.

Post-operative course

Post-operatively, she continued to have no motor or sensory deficits. Her reflexes were unchanged and she continued to void without difficulty. She was fitted for a custom lumbosacral orthotic (LSO) brace given her small stature. She wore the brace at all times and worked with physical therapy. Her operative drain was left until post-operative day two. Her pain was controlled with a combination of oxycodone, acetaminophen, and cyclobenzaprine. A tertiary trauma evaluation was performed which elucidated only incidental findings of bilateral renal cysts and left ovarian hemorrhagic cyst but no other traumatic injuries. She was discharged to home on post-operative day four. She was ambulating, taking adequate oral intake, and voiding independently without any urinary difficulty.

**Figure 4:**
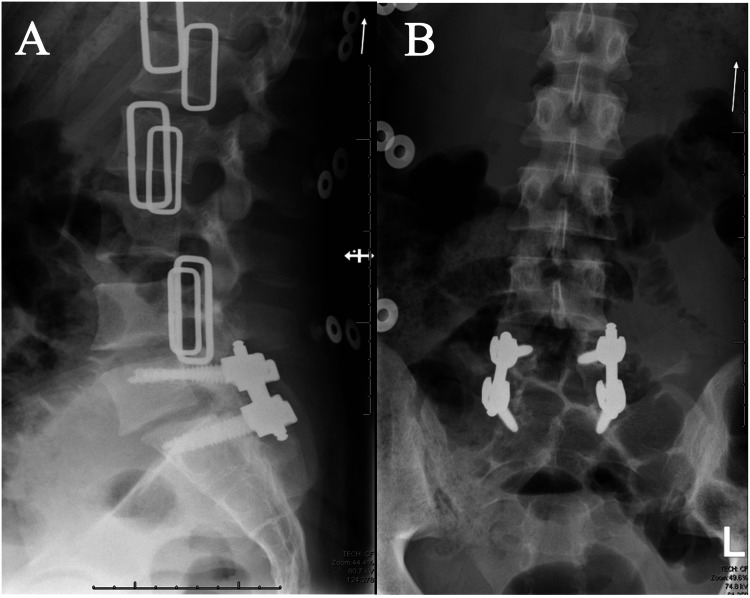
Post-operative anteroposterior and lateral lumbar spine x-rays (A) Lateral x-ray of the lumbar spine showing near anatomical post-operative reduction of the L5/S1 traumatic dislocation and spondylolisthesis with L5/S1 posterior-lateral pedicle screw instrumented fusion and (B) anteroposterior x-ray of the lumbar spine showing successful L5/S1 posterior-lateral pedicle screw instrumented fusion.

At her two-week follow-up visit, she continued to be neurologically intact and was having significant improvement in her back pain only intermittently requiring non-opioid pain medication. She stayed in an LSO brace for nine weeks. At that time, plain radiographs were obtained demonstrating stable alignment and intact hardware (Figures 4A, 4B).

## Discussion

Lumbosacral facet dislocation is a rare lesion typically seen in trauma and the pediatric population almost exclusively with high-energy accidents [8,9]. This type of injury is a severe flexion-distraction injury between vertebrae that occurs when the inferior articulating process of the superior vertebra is displaced anteriorly from the superior articular process of the inferior vertebra [10]. Jumped facets in the lumbar spine are rare, likely due to the combination of larger vertebral bodies and robust paraspinal musculature making it more difficult to dislocate the facets [11]. The majority of documented lumbar facet dislocation is reported at the L5-S1 junction, which is due to the facet joints being in the most coronal orientation with respect to the remaining lumbar facet joints [12,13]. A recent case series proposing a new classification for lumbosacral injuries would classify the present case into a 1C, meaning a bilateral dislocation with anterior slippage of the L5 vertebra and suggesting a flexion/distraction force [5]. This type of injury is the rarest and frequently associated with delays in diagnosis. What is notable about the present case is that there are only six reported cases of class 1C injuries, meaning pure dislocations without fractures of the articulating processes, pars interarticularis, or vertebral bodies (Table 1). In the present report, we discuss the third-ever reported class 1C injury in a pediatric patient and review the associated literature.

**Table 1:** Reported cases of class 1C lumbrosacral facet dislocation. MVC: motor vehicle collision; LLE: left lower extremity; RLE: right lower extremity; PD: posterior discectomy; PLIF: posterior lumbar interbody fusion; RTOR: return to operating room; PEEK: polyetheretherketone; BMP: bone morphogenetic protein; EHL: extensor hallucis longus; EDL: extensor digitorum longus; PF: plantar flexor

Reference	Publication year	Age (years)	Sex	Injury	Neurological exam on presentation	TP fracture	Disc disruption	L5 listhesis	Time to treatment	Surgery details	Follow up length	Neurological examination at follow-up
Aihara et al. [1]	1998	25	M	Crushed sitting between elevator and ceiling	Bowel/bladder dysfunction	Yes; multiple	Yes	50%	24 weeks	PD/PLIF w pedicle screws, RTOR 5 weeks later, L5/S1 discectomy	4 years	Unchanged bowel/bladder dysfunction
Beguiristain et al. [2]	1995	5	M	Hit in lumbosacral region with chair while knees flexed on floor	Left L5 sensory deficit, 0/5 and sensory deficit in left S1	Yes; multiple	Not reported	57%	immediately	No surgery	8 years	Normal
Dewey and Browne [6]	1968	31	F	MVC; back-seat passenger, no mention of seatbelt	S1 sensation loss, R foot weakness, bl paresthesias, urinary retention	yes; multiple	Not reported	50%	6 months	No surgery	6 months	Waddling gait, restricted movement; 3/5 LLE, 1/5 RLE.
Gertzbein [7]	1990	19	F	MVC; restrained passenger	4/5 RLE, diminished perineal sensation, decreased rectal tone	Not reported	Yes	>50%	immediately	Internal fixator through pedicles at L5 and S1 with tension-band around spinous processes, facet screws, and intertransverse fusion of L5/S1	2 years	RLE EHL, PF 4/5; LLE everters/EHL 0/5, PF 4/5
Verlaan et al. [3]	2001	17	M	Crushed flexed between forklift truck and metal bar	Left foot extensors weakness	Yes, L4	Yes	50%	8 days	L5-S1 laminectomy, durotomy repaired, pedicle fixation at L5/S1 with facetectomy, discectomy	1 year	Normal
Xu et al. [4]	2011	23	M	Hit in lumbosacral region with work machine while flexed forward	Left lower paresthesias, urinary retention. 4+/5 bl LE	Not reported	Yes	50%	48 hours	L5/S1 bl pedicle screw fixation with PEEK interbody device, bl lam/foram/facetectomy/discectomy for reduction.	1 year	normal
Santangelo et al. (this study)	2022	16	F	MVC; restrained driver	Normal	Yes; L5	Yes	50%	12 hours	L5-S1 laminectomy, facetectomy, discectomy, and pedicle screw fixation without interbody device	3 months	Normal

In reviewing the previously reported class 1C dislocations, the mechanism responsible is hyperflexion with force on the lumbosacral junction, consistent in all the other patients experiencing this type of injury. As seen in our case, injury to the L5-S1 disk occurs more readily in a seated or flexed position, as in a motor vehicle accident, as the hip joint is flexed and the lumbar spine is in lordosis allowing shear forces to act on the disk [6,9]. This shear force in combination with the hyperflexion is most likely what allows for the dislocation without fracture pattern [4].

Interestingly, two of the previously reported cases are in the pediatric population and all patients were equal to or less than 31 years of age [4]. Children typically have more ligamentous laxity which accounts for why only 2-3% of spinal fractures or dislocations occur in the pediatric population [9]. Fortunately, in our patient the lumbar neurocentral synchondrosis cartilaginous growth plates have already ossified so she is at less risk for impaired growth post-operatively. The range of patients experiencing a class 1C injury was 5 years to 31 years old, with three of six patients between 16 and 19 years of age. There is no male or female predominance seen in this injury [1-4,6,7].

Our case is different from prior traumatic lumbosacral dislocations reported in that the patient presented with a normal neurological examination. Of those reviewed in the present report, she is the only patient without a motor or sensory deficit or bowel/bladder dysfunction [1-4,6,7]. Similar to the others, she presented with severe low back pain. Also, our patient was without other traumatic injuries, which is unique in our case compared to others reported in a recent series of lumbosacral dislocations of varying classes who nearly all had other orthopedic or intra-abdominal injuries [5]. Unfortunately, in the cases of class 1C dislocations, this clinical information is only available for two cases, one having significant intra-abdominal injuries and the other having no other reported injuries [2,7].

In the reported case, non-contrasted CT demonstrated a transverse process fracture which is noted in the literature to be a “sentinel” sign of a lumbosacral injury [9,14]. Five of seven cases reported an associated transverse process fracture within the class 1C injuries and those that made no mention of this finding. Additionally, the presence of acute anterolisthesis of L5 on S1 suggests an L5-S1 disk disruption which we encountered in our case [5]. In reviewing the literature on dislocations without fracture, the degree of anterolisthesis was approximately 50%, or a grade II/III in all six cases, including the present report [15].

To the best of our knowledge, only five cases have been reported of traumatic dislocations of the lumbar spine in the pediatric population that were surgically treated [9,16-18]. Most commonly these were corrected with pedicle screw fixation and involved concurrent pars interarticularis or articulating process fractures, which is also true in the adult series of lumbosacral dislocations but there is also a report in the pediatric population using sublaminar cables for fixation [5]. Our patient underwent bilateral laminectomy with extensive facetectomy, discectomy, and pedicle screw fixation at the L5-S1 level. Of the class 1C injuries, four underwent pedicle fixation with facetectomy and discectomy including the present case [1,3,4]. One patient similarly underwent L5-S1 bilateral pedicle screw fixation with a tension band around the spinous processes and facet screws to facilitate intertransverse fusion of L5-S1 [7].

Case reports have varied in recommendation for including interbody fusion when there is the presence of disk disruption at the L5-S1 level, where some are of the opinion that with anatomic re-alignment and the integrity of the remaining disk that the anterior column has enough support [3,5,19]. In this case, we elected for posterior-only fusion given the patient’s age and robust nature of the remaining disk. However, it is clear that bilateral dislocations are associated with neurologic injury in up to one-third of all reported cases and should be surgically reduced and stabilized [4,20].

In the series of cases of class 1C injuries, all but two were definitively treated with surgery. The two cases that did not rely on traction are as follows: one patient was maintained in traction for two weeks and the other was cast in a reduced position for three months. The latter patient had no neurological deficits on follow up but the former at six months follow-up continued with restricted movement, gait disturbance, and hypoesthesia in the bilateral lower extremities. Among the cases of class 1C dislocations, three of seven patients were put into a brace; one of these was in lieu of surgical reduction and fixation [2,3]. In a recent case series including two adolescents, only two of 11 patients were not prescribed a brace post-operatively, the majority having an orthotic for typically three but up to six months [5]. Similarly, our patient was maintained in a custom LSO brace for nine weeks. Given the rarity of the condition and paucity of standard treatment guidelines, the decision for this timeframe was made based on clinical judgment. 

All patients in the review of class 1C injuries were followed with plain radiographs with follow-up time ranging from six months to eight years. At follow-up, three of seven continued to have deficits, although improved from the presentation and the remaining patients were neurologically intact. Only two patients continued to report pain requiring medication at follow-up [1-4,6,7].

## Conclusions

Class 1C lumbosacral facet dislocations are rare entities usually resulting from high-energy trauma. This report compares the presentation and surgical management of a 16-year-old female post-MVA with a class 1C lumbosacral dislocation to the six cases previously documented in the literature. It is the third case reported in a pediatric patient and the only case with normal neurological examination findings on presentation. This rare injury requires both thorough imaging assessments and an overall comprehensive clinical examination in order to accurately diagnose and subsequently treat the afflicted patient. After evaluation of our patient, she was successfully managed surgically with bilateral laminectomy, extensive facetectomy, discectomy, pedicle screw fixation, and a posterior-only interbody fusion. She was maintained in an LSO brace post-operatively. While the interventions used mainly paralleled those found in the literature, appropriate modifications were made to specifically address our patient’s needs. On follow-up, the patient remained neurologically intact and had significant improvement in her back pain.
